# Microstructure and Properties of Mg-Zn-Y Alloy Powder Compacted by Equal Channel Angular Pressing

**DOI:** 10.3390/ma11091678

**Published:** 2018-09-11

**Authors:** Chun Chiu, Hong-Min Huang

**Affiliations:** Department of Mechanical Engineering, National Taiwan University of Science and Technology, Taipei 106, Taiwan; m10403514@mail.ntust.edu.tw

**Keywords:** magnesium-yttrium-zinc alloy, mechanical milling, equal channel angular pressing, powder metallurgy

## Abstract

Mg_97_Zn_1_Y_2_ (at %) alloy with a long period stacking ordered (LPSO) phase has attracted a great deal of attention due to its excellent mechanical properties. It has been reported that this alloy could be fabricated by warm extrusion of rapid solidified alloy powders. In this study, an alternative route combining mechanical milling and equal channel angular pressing (ECAP) was selected to produce the bulk Mg_97_Zn_1_Y_2_ alloy. Microstructural characterization, mechanical properties and corrosion behavior of the ECAP-compacted alloys were studied. The as-cast alloy contained α-Mg and LPSO-Mg_12_Zn_1_Y_1_ phase. In the as-milled powder, the LPSO phase decomposed and formed Mg_24_Y_5_ phase. The ECAP-compacted alloy had identical phases to those of the as-milled sample. The compacted alloy exhibited a hardness of 120 HV and a compressive yield strength of 308 MPa, which were higher than those of the as-cast counterpart. The compacted alloy had better corrosion resistance, which was attributed to the reduced volume fraction of the secondary phase resulting in lower microgalvanic corrosion in the compacted alloy. The increase in Y content in the *α*-Mg matrix also contributed to the improvement of corrosion resistance.

## 1. Introduction

In the recent years, developments of magnesium alloys have increased dramatically due to the demands for light-weight structural materials with high specific strength to weight ratio and recyclability in automotive and aerospace industries [1,2,3,4]. However, the conventional AZ (Mg-Al-Zn), AM (Mg-Al-Mn) and ZK (Mg-Zn-Zr) series Mg alloys will not satisfy the extended industrial application for load-bearing components [5]. Therefore, it is needed to improve the performance of the conventional Mg alloys or to develop high-performance Mg alloys to meet the requirements. Among the newly developed Mg alloys, WZ (Mg-Zn-Y) series Mg alloy containing a long period stacking ordered (LPSO) structure has attracted much attention due to its excellent mechanical properties. Kawamura et al. fabricated WZ73 (90.7Mg-6.8Y-2.5Zn in wt %; Mg_97_Zn_1_Y_2_ in at %) alloy by warm extrusion of gas-atomized Mg_97_Zn_1_Y_2_ powders [6,7]. The bulk alloy prepared by rapidly solidified powder metallurgy (RS/PM) method exhibited an ultra-high tensile strength of 610 MPa and modest elongation of 5%. The high strength was attributed to the fine Mg matrix with a grain size of 100–200 nm and the presence of the LPSO phase.

Alternatively, bulk alloy can be produced by equal channel angular pressing (ECAP) of mechanically-milled powders. Mechanical milling (MM) is a solid-state powder processing technique, in which the welding, fracturing and rewelding of powders are repeated in a high-energy ball mill [8]. Non-equilibrium phase, super-saturated solid solution, as well as ultrafine or even monocrystalline grain have been obtained by mechanical milling process. Zhou et al. synthesized nanocrystalline AZ31 (Mg-3Al-1Zn in wt %) Mg alloy with titanium by mechanical milling [9]. After milling for 110 h, a nanocrystalline Mg matrix with a crystallite size of 66 nm was formed. Bulk alloy ingot was obtained by cold-pressing of as-milled powders. It was reported that as-milled AZ31 Mg alloy with 27 wt % Ti had a hardness of 147 HV, which was three times higher than that of the as-cast AZ31 Mg alloy. Koch et al. prepared nanocrystalline Mg_97_Zn_1_Y_2_ alloy powder by mechanical alloying of pure Mg, Zn and Y powders for 8 h [10]. The as-milled powder contained *α*-Mg and Mg_24_Y_5_ phase. Crystallite size of the two phases were 27 nm and 12 nm, respectively. LPSO phase was not formed after milling. Matsuda et al. synthesized Mg_97_Zn_1_Y_2_ powder by mechanical alloying of MgH_2_, Zn and Y powders [11]. It was found that MgH_2_ solid solution with Zn and Y was formed during the milling process and the grain of MgH_2_ was reduced to less than 30 nm. The bulk alloy produced by extrusion of the dehydrogenated powders contained *α*-Mg, MgO and Y_2_O_3_. The compressive yield strength of the bulk alloy was 616 MPa, which was higher than those of AZ91 (Mg-9Al-1Zn in wt %) and ZK60 (Mg-6Zn-0.5Zr in wt %) Mg alloys; however, the plastic strain was only ~4%. The enhanced strength was due to the grain refinement of Mg, solution hardening of Zn and Y in Mg and dispersion strengthening of MgO and Y_2_O_3_. LPSO phase was not observed in the as-milled powder or as-extruded bulk alloy when Mg (or MgH_2_), Zn and Y powders were used as starting materials. Thus, as-cast Mg_97_Zn_1_Y_2_ alloy containing LPSO phase should be considered as a starting material for mechanical milling.

Equal channel angular pressing (ECAP) is one of the severe plastic deformation (SPD) process used for obtaining ultrafine structures. To expand the power of ECAP to obtain nanometer grains, it has been suggested that powders, instead of bulk ingots should be used as starting material. Thus, ECAP processing for the powder was developed. In addition to warm extrusion process, bulk alloy or composites have been prepared by consolidation of alloy or ceramic reinforced metal matrix composite powders using ECAP [12,13,14,15,16]. It has also been shown that it was possible to produce compacted bulk samples with nanostructures. Karman et al. demonstrated that bulk Cu material with grain size less than 100 nm could be obtained by ECAP [17]. Recently, Lee et al. investigated the consolidation of Mg powders using ECAP [18]. When the ~200 mesh grade Mg powder was used as the staring material, the consolidated bulk material after 4 passes of ECAP at 300 °C exhibited the best properties. The XRD-calculated grain size (grain size calculated by X-ray diffraction) was 92.8 nm and the ultimate compressive strength and compressive yield strength were 193 MPa and 100 MPa, respectively. There is no research on the properties of bulk Mg-Zn-Y alloy compacted from as-cast Mg-Zn-Y alloy powders using ECAP.

Literature reviews revealed that no prior attempts have been made to combine the mechanical milling and ECAP to produce compacted Mg-Zn-Y alloys. Accordingly, the aim of the present study is to fabricate bulk Mg_97_Zn_1_Y_2_ alloy by ECAP of milled Mg_97_Zn_1_Y_2_ powders and to provide and early insight on the properties of the compacted bulk alloy. In the present study, as-cast Mg_97_Zn_1_Y_2_ alloy was used as starting material for mechanical milling. The effect of number of ECAP pass on the consolidation condition of powders was investigated. Moreover, microstructure, mechanical properties and corrosion behavior of the bulk Mg_97_Zn_1_Y_2_ alloy prepared by ECAP of milled powders were also studied.

## 2. Materials and Methods

Mg_97_Zn_1_Y_2_ (at %) alloy was fabricated by melting pure Mg (99.98 wt %), Zn (99.9 wt %) and Y (99.9 wt %) ingots in an electric resistant furnace (homemade) under the protection of a mixture gas (99% CO_2_ and 1% SF_6_) at 750 °C. The starting materials used in the present study were all commercially purchased. Mg ingot was purchased from Mach Taiwan Technology Co., Ltd. (Taipei, Taiwan), while Zn and Y ingots were purchased from Alfa Aesar (Heysham, UK). After mechanical stirring at 400 rpm for 15 min, the molten alloy was poured into a steel mold preheated at 200 °C and cooled in air. Ingot chips were obtained by cutting as-cast ingot using a vertical milling machine. Mg_97_Zn_1_Y_2_ alloy powder was prepared by mechanical milling of as-cast ingot chips for 40 h under argon in a planetary ball mill (PM110, RETSCH, Haan, Germany). The rotation speed and ball-to-powder ratio are 300 rpm and 25, respectively. To prevent cold welding of powders, 2 wt % of toluene was used as process control agent. All the powder handling was performed in a glove box (ASONE International, Inc., Santa Clara, CA, USA) to prevent oxidation.

For ECAP process, a copper tube having outer dimensions of 19 mm × 19 mm × 75 mm and inner powder chamber with dimensions of 12 mm in diameter and 65 mm in depth was used. The copper tube was filled with the as-milled powders, compacted by mechanical force and sealed with a copper cup. The sealed tubes were then processed by ECAP. Samples were extruded for 1, 2 and 4 passes in route B_c_ (rotating the sample around its longitudinal axis after each pass by 90° clockwise) at 300 °C. The pressing was performed in a die having an internal channel angle *Φ* = 120° and an angle at the outer arc of curvature of the two parts of the channel *Ψ* = 60° at a constant ram speed of 1.0 mm/s and temperature of 300 °C. With these angles, a strain of ~0.7 was produced for each pass through the die. The die was heated to 300 °C by a collar heating band and a thermocouple attached near the cross section of the die provided the control of temperature within ±5 °C. Before pressing, the billets were inserted into the preheated-die and keep for 10 min to allow temperature equilibration.

Microstructural characterization of the samples was conducted with an optical microscope (OM, OLYMPUS, Tokyo, Japan) and a field-emission scanning electron microscope (FE-SEM) (JSM-6500F, JEOL, Tokyo, Japan) equipped with Energy Dispersive Spectroscopy (EDS, Oxford Instrument, Abingdon, UK). At least five readings were made from which the mean value were calculated. Area fraction of secondary phase was estimated using Image J 1.51K software. The constituent phases in the samples were examined by X-ray diffraction (XRD) (D2 PHASER, Bruker, Madison, WI, USA) using Cu K*α* radiation with the scan range of 20° to 90°. The data were collected with a step size of 0.02° and time of 0.5 s. LPSO phase was identified using the XRD pattern of as-cast Mg_97_Zn_1_Y_2_ alloy reported by Yamasaki et al. [19]. The crystallite size of Mg in the powders and bulk alloys was calculated by Scherrer equation. Differential Scanning Calorimetry (DSC) analysis was performed using STA449F3 (Netzsch, Selb, Germany). About 15 mg of sample was placed in an alumina sample pan and was heated from room temperature up to 680 °C under an argon flow rate of 20 mL/min with a heating rate of 5 °C/min. Particle size distribution of the as-milled powders was measured by a laser particle size analyzer (Mastersizer 2000, Malvern Panalytical, Malvern, UK).

Density measurements of the compacted samples were performed after removing the surrounding Cu tube. Experimental density of the sample (*ρ*_E_) was measured by Archimedes method; while the theoretical density (*ρ*_T_) was estimated using rule of mixture. The porosity (*ϕ*) of the sample was calculated from the difference between the theoretical and experimental porosity [20]:

*ϕ* = 1 − *ρ*_E_/*ρ*_T_ (1)

Room-temperature compression test was performed using a universal material testing machine MTS810 (MTS, Eden Prairie, MN, USA). Samples with a size of 5 mm × 5 mm × 5 mm were tested under a constant strain rate of 10^−3^/s. Vickers microhardness tests were performed using Akashi MVK-H1 microhardness tester (Mitutoyo, Kawasaki, Japan) with a load of 100 g for 15 s dwell time. At least ten readings were made from which the mean value were calculated.

Corrosion performance was analyzed by immersion test. Immersion tests were conducted in 3.5 wt % NaCl solution at room temperature for 24 h. Approximately 60 mL solution per cm^2^ specimen surface was used during the test. Surfaces of rectangular samples with a dimension of 7 mm × 7 mm × 3 mm were ground with SiC sandpaper up to 2500 grit, then ultrasonically cleaned in alcohol and dried. After immersion periods of 1, 12 and 24 h, the samples were removed from the solution, cleaned and dried. The corrosion surfaces of samples were observed using a digital camera and a SEM. The composition of corrosion products was analyzed by EDS and XRD. Thereafter, the corrosion products on the samples were removed by submerging the samples in a chromic acid bath (180 g/L CrO_3_). The samples were then rinsed with deionized water and dried. Finally, the masses of samples were recorded.

## 3. Results and Discussion

### 3.1. Microstructural Characterization

#### 3.1.1. As-Cast Mg_97_Zn_1_Y_2_ Alloy

**Figure 1:**
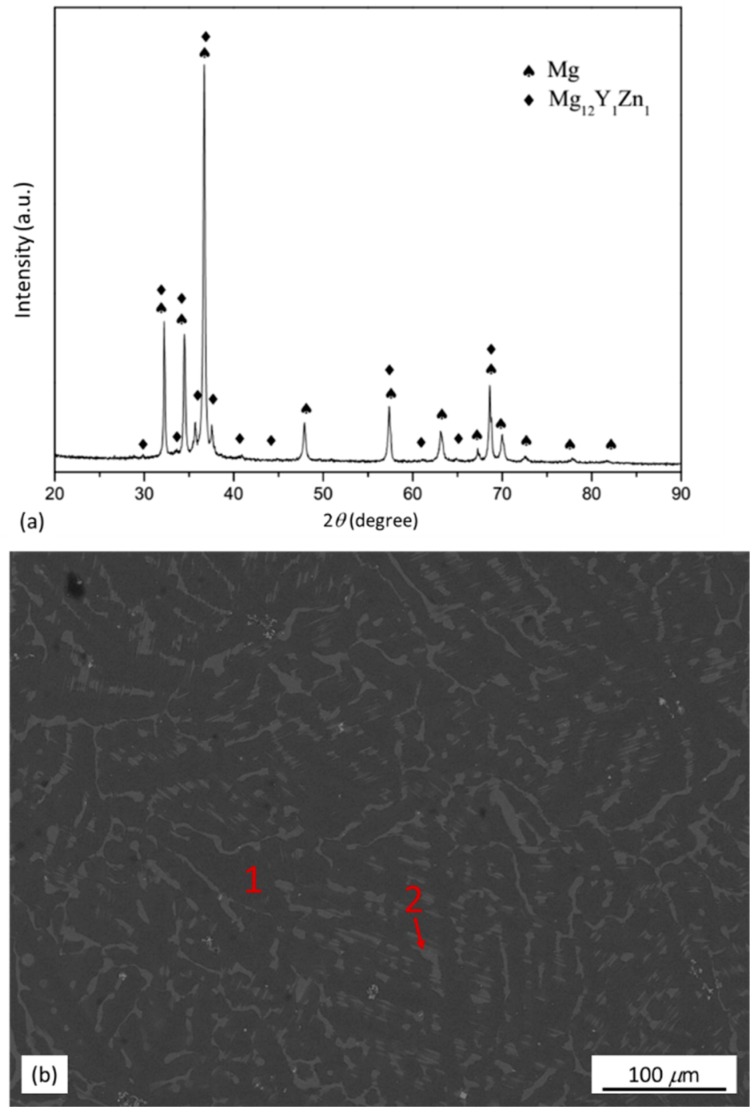
(**a**) XRD pattern and (**b**) BSE-SEM micrograph of as-cast Mg_97_Zn_1_Y_2_ alloy.

XRD pattern and back scattered electron (BSE)-SEM micrograph of the as-cast Mg_97_Zn_1_Y_2_ alloy are shown in Figure 1. From the XRD pattern in Figure 1a, it was identified that the as-cast alloy was composed of *α*-Mg and Mg_12_Y_1_Zn_1_ phase. The Mg_12_Y_1_Zn_1_ phase in the as-cast alloy had a 18R-type LPSO structure reported by Yamasaki et al. [19]. To further identify the distribution of the phases, EDS analysis was performed on selected areas in Figure 1b. The chemical compositions given in Table 1 indicated that zone 1 (matrix) was *α*-Mg solid solution with a relatively low content of Zn and Y and zone 2 (secondary phase) was a phase with a relatively high content of Zn and Y, which could be assigned to Mg_12_Z_1_Y_1_ (at %) phase with a LPSO structure as reported in Refs [5,19,21,22,23] (Table 1).

#### 3.1.2. As-Milled Mg_97_Zn_1_Y_2_ Powders

**Figure 2:**
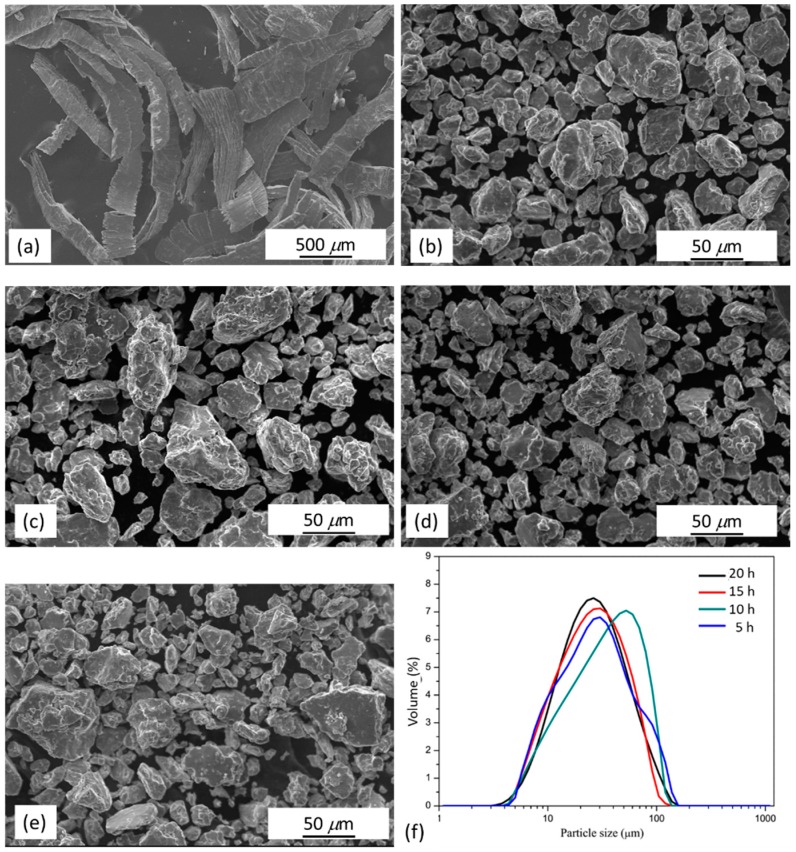
SEM micrographs of (**a**) Mg_97_Zn_1_Y_2_ alloy chips; Mg_97_Zn_1_Y_2_ alloy after milling for (**b**) 5 h, (**c**) 10 h, (**d**) 15 h and (**e**) 20 h; (**f**) particle size distribution of as-milled powders.

Mg_97_Zn_1_Y_2_ alloy chips having dimensions of several millimeters were used as starting materials for mechanical milling (Figure 2a). After milling for 5 h, the morphology of the sample transformed from laminar chips to equiaxed particles with an average particle size of 38 μm (Figure 2b). Further increase of milling time did not alter the morphology of particles (Figure 2c–e). As shown in Table 2 and Figure 2f, when the milling time increased, the particle size remained almost constant. XRD-calculated crystallite sizes were in the range of 22 to 24 nm. This indicated the equilibrium of fracturing and welding in the milling process has been reached after milling for 5 h.

**Figure 3:**
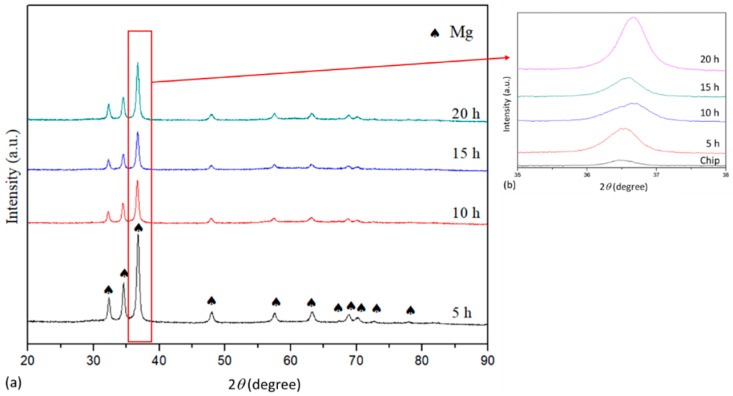
XRD patterns of (**a**) Mg_97_Zn_1_Y_2_ alloy after milling for 5 h, 10 h, 15 h and 20 h; (**b**) zoom-in pattern from 36° to 38° showing peak shifting of Mg.

**Figure 4:**
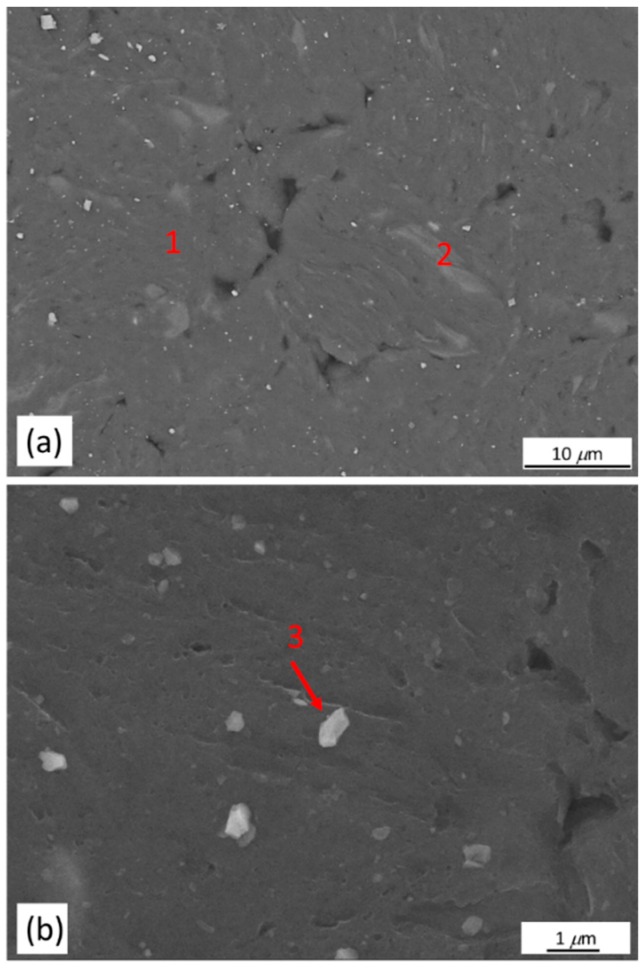
(**a**) BSE-SEM micrograph of 5 h-milled Mg_97_Zn_1_Y_2_ alloy; (**b**) higher magnification SEM micrograph showing dispersion of particles.

Evolution of phase during the milling process was investigated using XRD analysis. As shown in Figure 3a, only peaks belonged to Mg phase were observed in XRD pattern, indicating the LPSO-Mg_12_Zn_1_Y_1_ phase either decomposed or went through a fine refinement so that it was not detected by XRD after milling for 5 h. No areas having chemical composition of LPSO-Mg_12_Zn_1_Y_1_ phase was observed in the as-milled powders by SEM-EDS analysis (Figure 4 and Table 1). Further increasing milling time resulted in gradually shifting of peaks to higher diffraction angle, implying the lattice parameter of Mg decreased with the increase of milling time (Figure 3b). This was due to the reason that more Zn and Y dissolved in Mg and formed super saturated solid solution as milling time increased, which was commonly observed during a milling process.

No new intermetallic phases were detected by XRD. However, as shown in Figure 4 and Table 1, SEM-EDS indicated formation of new phases or microconstituents after milling. In the 5 h-milled sample, dark gray *α*-Mg matrix (zone 1) was a Mg(Zn,Y) solid solution having 1.18 at % of Zn and 1.79 at % of Y. The amounts of Zn and Y were higher than those of *α*-Mg matrix in the as-cast alloy (0.58 at % Zn and 1.10 at % Y). Two zones which could be distinguished from the *α*-Mg matrix by their chemical composition and contrast (light gray contrast in zone 2 and white contrast in zone 3) were observed after milling for 5 h. The zone 2 had 1.15 at % of Zn and 4.87 at % of Y. Compared to the *α*-Mg matrix (zone 1), it was richer in Zn and Y (Figure 4a and Table 1). White particles (zone 3) having a composition of 84.80Mg-1.15Zn-14.05Y (at %) could be assigned to Mg_24_Y_5_ phase (Figure 4b). The particle size of Mg_24_Y_5_ was in the range of 200 to 700 nm. The reason that Mg_24_Y_5_ phase was not observed in the XRD pattern was because the amount of the phase was below the detection limit of XRD analysis.

According to ternary Mg-Zn-Y phase diagram, three types of Mg-Zn-Y phases, including I phase (Mg_3_Zn_6_Y_1_), W phase (Mg_3_Zn_3_Y_2_) and X phase (Mg_12_Zn_1_Y_1_) are commonly observed in the Mg-rich region [24,25]. From the ternary phase diagram, it has been shown that the formation of I, W or X phase depends on the Y/Zn ratio in Mg-Zn-Y alloys [26,27]. For the Mg_97_Zn_1_Y_2_ alloy investigated in the present study, Y/Zn ratio was 2 and the *α*-Mg and Mg_12_Zn_1_Y_1_ phase were presented in the as-cast alloy. XRD peaks belonging to I, W and X phases were not identified in the XRD patterns of the milled powder. Furthermore, from EDS analysis, the zone 2 in the 5 h-milled sample did not match the chemical composition of W, X and I phases. According to Mg-Y phase diagram, the solubility of Y in Mg could go up to 3.75 at % [28]. The Y content in zone 2 was above the solubility of Y in Mg. Given that mechanical milling was a non-equilibrium process, it was reasonable to expect that a super saturated solid solution with a higher Y content was formed after decomposition of LPSO phase. Thus, it was possible that the zone 2 was a supersaturated solid solution Mg(Zn,Y). However, as will be shown later in Section 3.1.3, zone 2 having 1.24 at % of Zn and 4.31 at % of Y, which was similar to that in the 5 h-milled powder, was still observed after ECAP at 300 °C. If the zone 2 represented a supersaturated solid solution, after processing at 300 °C, its Y content should be lower than he solubility of Y in Mg (~2 at %) at 300 °C [28]. However, the Y content in zone 2 (4.31 at %) was way above the solubility. Thus, the formation of supersaturated solid solution was unlikely.

**Figure 5:**
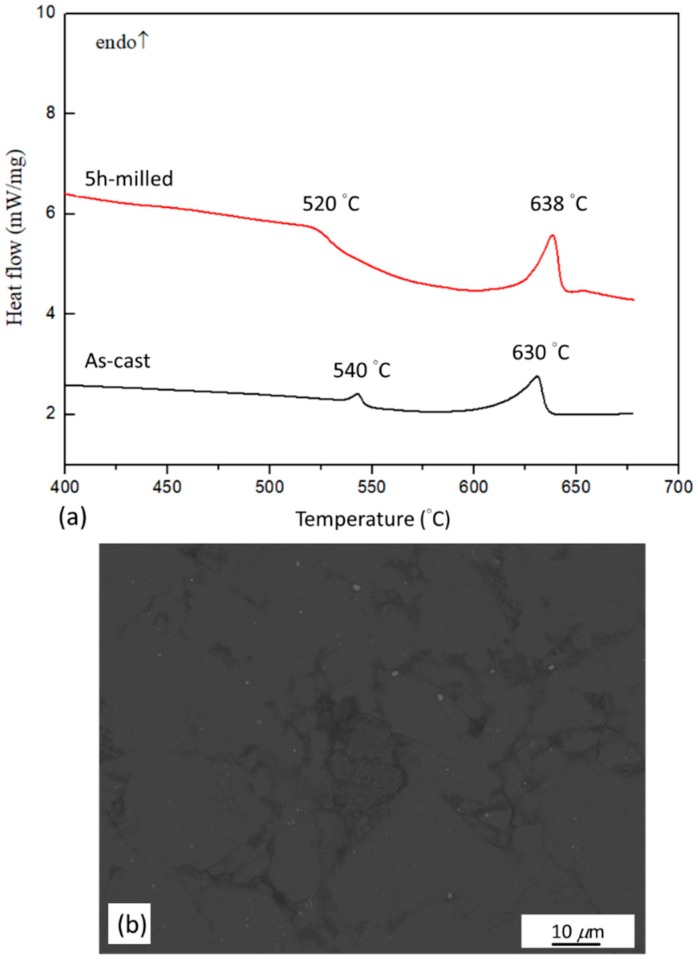
(**a**) DSC curves of as-cast Mg_97_Zn_1_Y_2_ ingot and 5 h-milled Mg_97_Zn_1_Y_2_ powder; (**b**) BSE-SEM micrograph of the 5 h-milled Mg_97_Zn_1_Y_2_ powder after DSC heating to 550 °C.

The disappearance of LPSO-Mg_12_Zn_1_Y_1_ phase on the XRD pattern after milling was further studied by DSC analysis. DSC curves of the as-cast Mg_97_Zn_1_Y_2_ ingot and 5 h-milled Mg_97_Zn_1_Y_2_ powder are shown in Figure 5a. Two endothermic peaks at 540 °C and 630 °C could be observed for the DSC curve of the as-cast ingot. The secondary peak correlated well with the melting of Mg. Results from XRD analysis indicated that there was only one secondary phase (LPSO-Mg_12_Zn_1_Y_1_) in as-cast alloy, which correlated well with the phases reported for Mg_97_Zn_1_Y_2_ in a Mg-Zn-Y ternary phase diagram [26]. As a result, the first peak in the DSC curve of the as-cast alloy was due to the dissolution of the LPSO phase, which was also reported by Su et al. and Chen et al. for DTA thermograms of Mg-Zn-Y alloys [5,27]. However, for the 5 h-milled alloy powder, besides a peak belonged to melting of Mg at 638 °C, a broadened and weak peak was observed at 520 °C. Figure 5b shows a BSE-SEM micrograph of the 5 h-milled powder after heating to 550 °C in a DSC test. It was observed that zone 2 with a light gray contrast disappeared. This indicated that the peak at 520 °C was due to the dissolution of zone 2. Assume retained LPSO phase existed in zone 2 after milling for 5 h, it could explain why zone 2 was still preserved after ECAP at 300 °C and decomposed at 520 °C in the DSC measurement.

Upon milling, the semi-continuous network of LPSO phase broke down due to the repeated fracturing. Zn and Y in the LPSO phase entered the *α*-Mg matrix and the LPSO phase decomposed. The excess Y in the matrix reacted with Mg and formed particulate Mg_24_Y_5_ intermetallic phase. Most likely, some LPSO phase still retained in the grain boundary region. The amount of retained LPSO phase was low and its structure was refined during the milling process, which made it difficult to be detected by XRD analysis.

Unlike the powder prepared by rapid solidification (RS) process, the LPSO phase was not preserved in the powder processed by ball milling process. The Mechanically driven decomposition or transformation of phases during a milling process has been reported. Gerasimov et al. found that Ti_3_Cu_4_ phase transformed into TiCu and TiCu_4_ [29]. Kwon et al. reported that the FeSn intermetallic compound decomposed with the formation of the Fe_5_Sn_3_ and FeSn_2_ phases during the milling process [30]. Thus, it was feasible for the decomposition of Mg_12_Zn_1_Y_1_ phase during the milling process.

#### 3.1.3. Mg_97_Zn_1_Y_2_ Powders Compacted by ECAP

**Figure 6:**
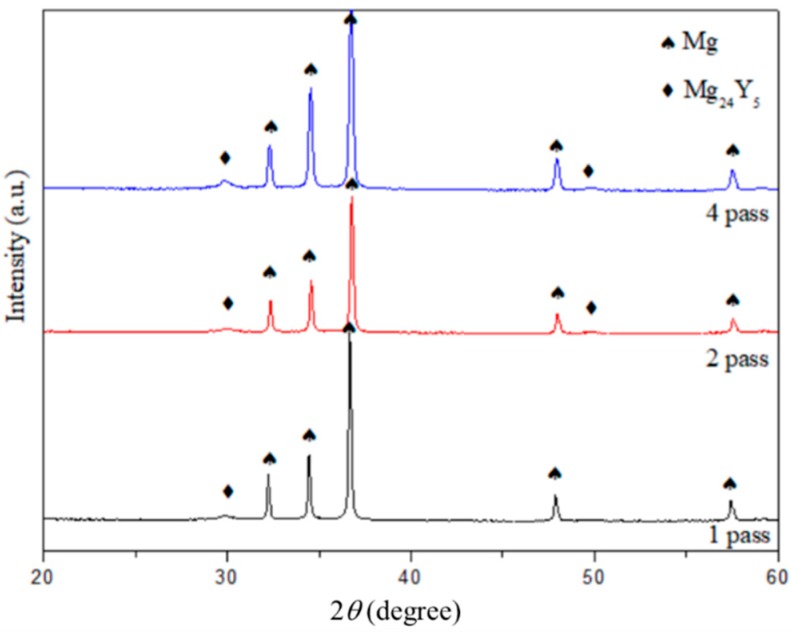
XRD patterns of Mg_97_Zn_1_Y_2_ powders after compacting by ECAP for 1, 2 and 4 passes.

Figure 6 shows the XRD patterns of Mg_97_Zn_1_Y_2_ powders after compacting by ECAP at 300 °C for 1, 2 and 4 passes. Regardless of the number of pass, the consolidated bulk samples contained *α*-Mg and Mg_24_Y_5_ phase. XRD-calculated crystallite sizes of Mg increased from 24 nm in the 5 h-milled sample to 57 nm in the sample after 1pass of ECAP and further decreased to 41 nm when the number of pass increased to 4 times. This indicated that grain growth after 1 pass of ECAP at 300 °C and grain refinement after 2 passes of ECAP, which agreed with the reported results that ECAP process is able to refine the grain size when the number of passes increases [31].

**Figure 7:**
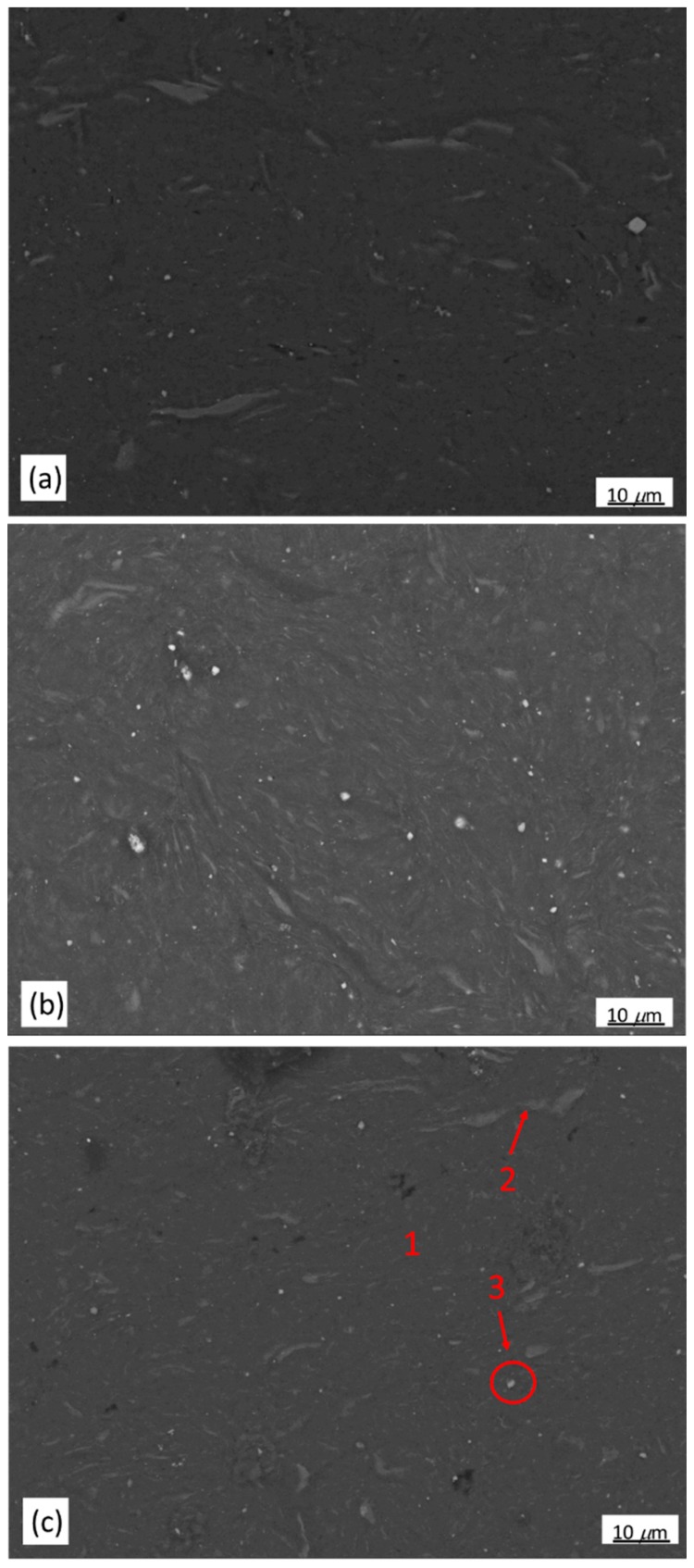
BSE-SEM micrographs of Mg_97_Zn_1_Y_2_ powders after compacting by ECAP for (**a**) 1; (**b**) 2 and (**c**) 4 passes.

Microstructures of the powder samples compacted by ECAP at 300 °C for 1, 2 and 4 passes are shown in Figure 7a–c. The BSE-SEM micrograph showing the selected areas for EDS analysis of the compacted bulk sample after 4 passes of ECAP is shown in Figure 7c. Three distinct zones similar to those of the as-milled powder could be observed in the micrograph. The dark gray zone 1 was *α*-Mg matrix, a solid solution containing lower amounts of Zn and Y; the light gray zone 2 was a retained LPSO phase with a refined structure; the particulate zone 3 has a composition very close to 82.76Mg-17.24Y (at %), which could be assigned to Mg_24_Y_5_ phase. Compared to the 5 h-milled sample, the composition of these three zones did not change after ECAP process, indicating no phase transformation occurred when the samples underwent to 4 passes. The major difference among the milled samples and samples after ECAP for 1,2 and 4 passes were the refinement of retained LPSO phase (zone 2) (Figure 4a and Figure 7a–c). ECAP effectively broke down the large retained LPSO phase into small ones during the process.

The density of the compacted samples increased with the increasing number of ECAP passes. As a result, porosity of the compacted sample decreased from 4.6% after 1 pass to 2.4% after 4 passes (Table 2). Compared to the reported porosity of powder samples prepared by other powder compaction method such as hot extrusion or spark plasma sintering, the porosity of 2.4% is too high to be considered for practical application. In the future study, the ECAP process should be improved (e.g., ECAP with back pressure) to reduce the porosity.

### 3.2. Mechanical Properties

Compared to the as-cast sample (80 HV), Vickers microhardness increased in the ECAP-compacted sample (~120 HV), which resulted from the finely dispersion of Mg_24_Y_5_ particles (Figure 6 and Figure 7). However, increasing number of ECAP pass did not improve the microhardness.

**Figure 8:**
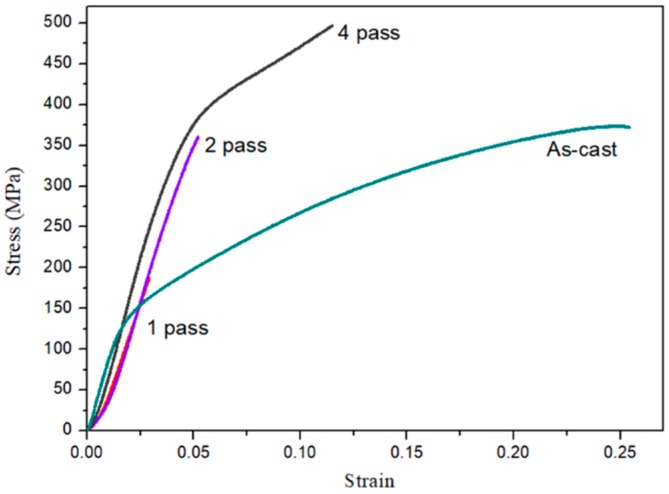
Compressive stress-strain curves of as-cast Mg_97_Zn_1_Y_2_ and compacted Mg_97_Zn_1_Y_2_ samples produced by ECAP of Mg_97_Zn_1_Y_2_ powders for 1, 2 and 4 passes.

Figure 8 shows results of compressive tests for as-cast Mg_97_Zn_1_Y_2_ and compacted Mg_97_Zn_1_Y_2_ after 1, 2 and 4 passes of ECAP at 300 °C. Due to the brittle behavior of the compacted samples after 1 and 2 ECAP passes, only the compressive yield strength (CYS) of the compacted sample after 4 ECAP passes was used to evaluate the strength of the ECAPed sample. After 4 passes of ECAP, CYS of the compacted sample reached 308 MPa and failure strain increased to 0.11. By contrast, CYS and failure strain of the as-cast alloy were 146 MPa and 0.25, respectively. It has to be pointed out that the length to width ratio of the specimen used for compression test was less than 1.5. An overestimation of CYS might occur.

It is well known that the grain size of the matrix, particle size of dispersed phase and porosity are important factors that affect the compressive stress of the compacted samples. Lee et al. studied the mechanical properties of the consolidated Mg powders prepared by equal channel angular extrusion [18]. It was found that the CYS increased with the decreasing size of oxide particles in the compacted samples. In the present study, increased solubility of Zn and Y in Mg matrix and finely dispersed Mg_24_Y_5_ particles were clearly observed in the compacted Mg_97_Zn_1_Y_2_ sample. Enhancement of CYS was expected for the compacted sample produced by ECAP due to solid solution strengthening and dispersion strengthening. The sample after 4 ECAP passes possessed the finest crystallite size and the lowest porosity. Thus, the CYS of the sample after 4 ECAP passes was effectively enhanced but the ductility was sacrificed as compared to the as-cast Mg_97_Zn_1_Y_2_ sample.

### 3.3. Corrosion Behavior

**Figure 9:**
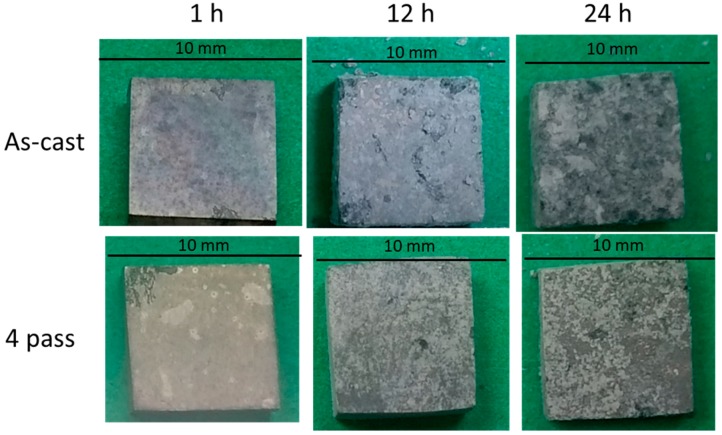
Marco corrosion morphologies of the as-cast Mg_97_Zn_1_Y_2_ and EP4-Mg_97_Zn_1_Y_2_ after immersing in 3.5 wt % NaCl solution for 1, 12 and 24 h.

The corrosion behavior of the as-cast Mg_97_Zn_1_Y_2_ and compacted Mg_97_Zn_1_Y_2_ prepared by 4 ECAP passes (designated in the text as EP4-Mg_97_Zn_1_Y_2_) was studied by immersion test. The evolution of macro corrosion morphologies of the as-cast Mg_97_Zn_1_Y_2_ and EP4-Mg_97_Zn_1_Y_2_ at different immersion times is shown in Figure 9. There was no huge difference between the corrosion surfaces of the as-cast Mg_97_Zn_1_Y_2_ and EP4-Mg_97_Zn_1_Y_2_ after immersing for 1 h. However, when the immersion time went beyond 12 h, it was clear that the corrosion was more sever in the as-cast sample.

**Figure 10:**
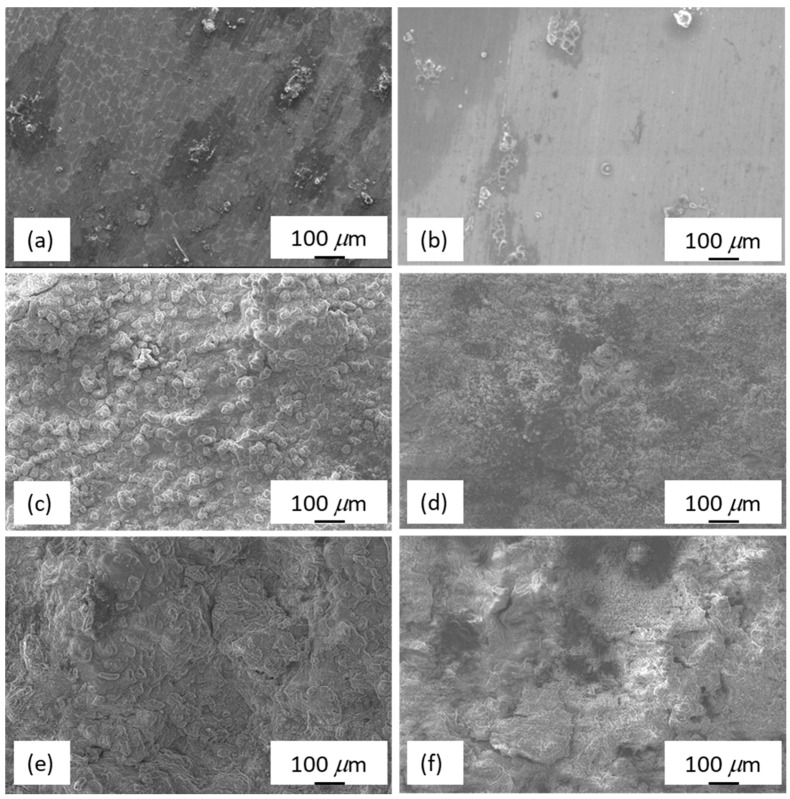
Surface morphology of the as-cast Mg_97_Zn_1_Y_2_ after immersing in 3.5 wt % NaCl solution for (**a**) 1; (**c**) 12 and (**e**) 24 h; surface morphology of EP4-Mg_97_Zn_1_Y_2_ after immersing in 3.5 wt % NaCl solution for (**b**) 1; (**d**) 12 and (f) 24 h.

**Figure 11:**
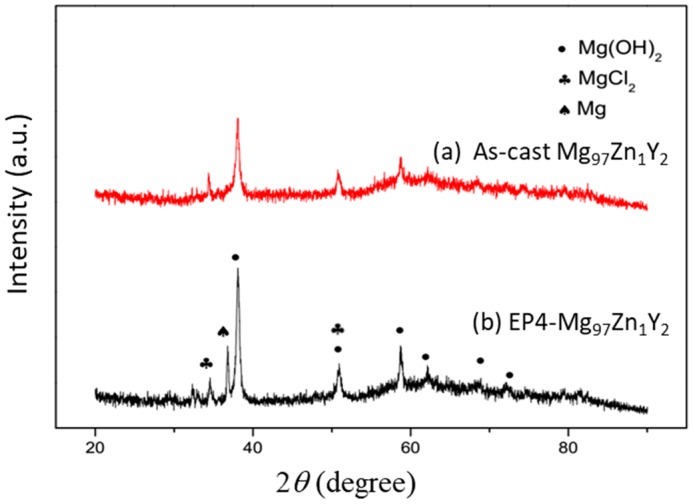
XRD patterns of the surface corrosion layers in (**a**) the as-cast Mg_97_Zn_1_Y_2_ and (**b**) EP4-Mg_97_Zn_1_Y_2_ after immersing in 3.5 wt % NaCl solution for 12 h.

Figure 10 shows the SEM surface morphology of the as-cast Mg_97_Zn_1_Y_2_ and EP4-Mg_97_Zn_1_Y_2_ after immersing in 3.5 wt % NaCl solution for 1, 12 and 24 h. After immersing for 1 h, the corroded areas were small and isolated, indicating the corrosion layer has just formed (Figure 10a,b). When immersion time increased, the corrosion layer grew and covered more surface areas. EDS results indicated corrosion products containing Mg, O and Cl (46.19 at % Mg-37.10 at % O-16.71 at % Cl for the as-cast one; 40.55 at % Mg-48.03 at % O-11.42 at % Cl for the EP4 one). XRD analysis confirmed that the corrosion products were Mg(OH)_2_ and MgCl_2_ (Figure 11), suggesting that corrosion reactions happened on the surface of Mg_97_Zn_1_Y_2_ were similar to those reported for Mg alloys immersed in solution containing Cl^−^ [32,33].

**Figure 12:**
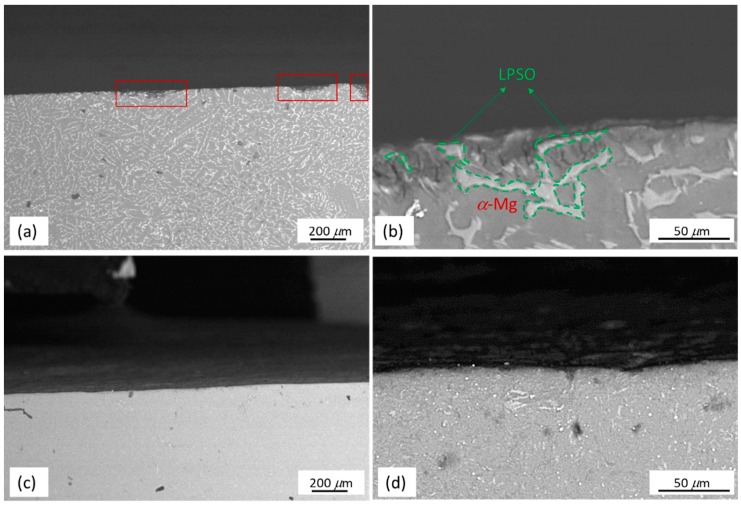
Cross sectional images of as-cast Mg_97_Zn_1_Y_2_ and EP4-Mg_97_Zn_1_Y_2_ samples after immersing in 3.5 wt % NaCl solution for 12 h; (**a**) low and (**b**) high magnification image of as-cast Mg_97_Zn_1_Y_2_; (**c**) low and (**d**) high magnification image of EP4-Mg_97_Zn_1_Y_2_.

Cross sectional images of the as-cast Mg_97_Zn_1_Y_2_ and EP4-Mg_97_Zn_1_Y_2_ after immersing in 3.5 wt % NaCl solution are shown in Figure 12. As shown in Figure 12a, corrosion pits (marked with red frames) were observed on the surface of the as-cast sample. A zoom-in image of the corrosion pit is shown in Figure 12b. It was found that *α*-Mg matrix was under more serous corrosion, indicating that the LPSO phase had a higher corrosion resistance than that of *α*-Mg matrix. The difference between the composition of the LPSO phase and *α*-Mg matrix resulted in potential difference and micro-galvanic corrosion was observed. In the galvanic corrosion, the LPSO phase acts as a micro-cathode and *α*-Mg matrix acts as a micro-anode. The initial corrosion started at the interface of the LPSO phase and *α*-Mg matrix. Similar corrosion behavior was also reported for Mg-Zn-Gd-Zr alloys and Mg-Y-Er-Zn alloys containing LPSO phase [34,35]. Compared to the as-cast Mg_97_Zn_1_Y_2_, no visible pits are observed on the surface of the EP4 sample (Figure 12c,d).

**Figure 13:**
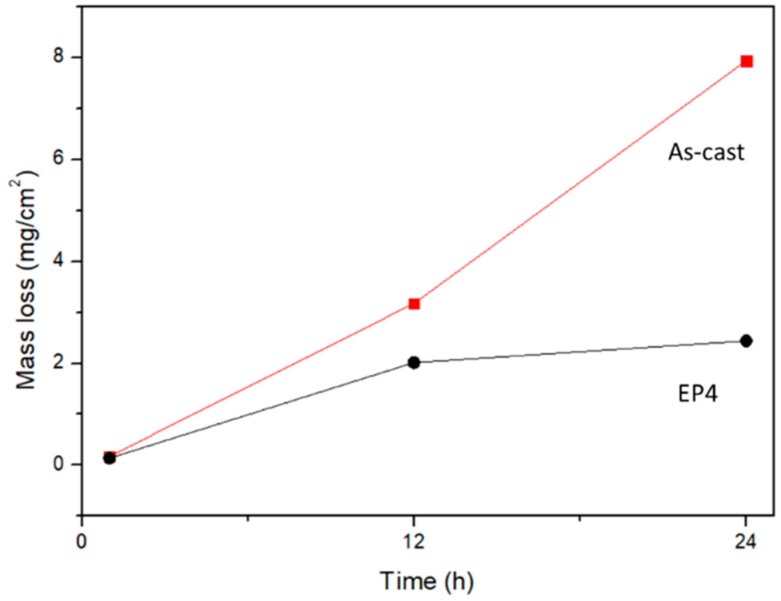
Mass losses of the as-cast Mg_97_Zn_1_Y_2_ and EP4-Mg_97_Zn_1_Y_2_ as a function of immersion time in a 3.5 wt % NaCl solution.

The mass losses of the as-cast Mg_97_Zn_1_Y_2_ and EP4-Mg_97_Zn_1_Y_2_ immersed for 1, 12 and 24 h in a 3.5 wt % NaCl solution are shown in Figure 13. Mass losses of the both samples increased with increasing immersing time. The mass loss of the as-cast sample showed a sharp increase, while that of the compacted showed a slower increase. The mass loss of the as-cast Mg_97_Zn_1_Y_2_ was higher than that of the EP4 sample, suggesting that corrosion resistance of the alloys was enhanced in the EP4 sample produced by ball milling and ECAP process.

Similar results that Mg alloys prepared via powder metallurgy route have improved corrosion resistance have been reported. Liao et al. compared the corrosion resistance of as-cast Mg-Al-Mn-Ca alloy (AMX602) and the same alloy after spinning water atomization process (SWAP) [36]. The corrosion resistance of the SWAPed alloy was 10 times higher than that of the as-cast one. The as-cast alloy contained coarse *α*-Mg matrix and netlike constituents of eutectic *α*-Mg and Al_2_Ca phase distributed along the grain boundaries, while the SWAPed alloy contained fine *α*-Mg grains and dispersed Al_2_Ca particles. Because of the refinement of microstructure, the ratio of cathode (Al_2_Ca particle) to anode (*α*-Mg) decreased. Thus, the microgalvanic corrosion between the Al_2_Ca and *α*-Mg was depressed. Kubásek et al. reported the corrosion properties of WE43 (Mg-Y-Nd) alloy prepared by powder metallurgy and concluded that the improvement of corrosion resistance of WE43 alloy prepared by powder metallurgy (WE43-PM) compared to that of WE43 prepared by casting and extrusion (WE43-IM) was attributed to the finer structure and homogeneously dispersed fine secondary phases, which resulted in the decreased cathode to anode ratio [37].

However, results showing detrimental effect of powder metallurgy processing on corrosion resistance also exist. Cabeza et al. compared corrosion behavior of WZ21 (Mg-2Y-1Zn in wt %) Mg alloys prepared by extrusion of a cast ingot (WZ21-IM350) and by extrusion of rapidly solidified alloy powders (WZ21-PM350) [38]. It was found that WZ21-PM350 had lower corrosion resistance. The fine homogenous dispersion of secondary-phase particles in WZ21-PM350 acted as numerous cathodic sites and promoted microgalvanic corrosion. Pérez et al. studied the corrosion behavior of Mg-Zn-Y-Mischmetal alloy (Mg_95_Zn_1.5_CeMM_1.5_ in at %, designated as MgRE_1.5_) [39]. Two types of alloy, MgRE_1.5_ and MgRE_1.5_-PM were fabricated by hot extrusion of as-cast alloy and gas-atomized alloy powder, respectively. Fine dispersion of secondary-phase particles was also observed in the MgRE_1.5_-PM. The particles accelerated the activity of microgalvanic cell and decreased the corrosion resistance.

The change of corrosion resistance can be attributed to the microstructural change brought by processing. The different corrosion behaviors observed in the as-cast/extruded alloy and the alloy prepared by powder metallurgy route were related to the amount, size, morphology and distribution of secondary phase, as well as the size of the Mg matrix because they determined the driving force of microgalvanic corrosion. It has been reported that the galvanic corrosion is strongly affected by the ratio of cathode to anode. When the structure of Mg matrix was refined and the secondary phase was refined and dispersed, the surface area ratio of cathode (secondary phase) to anode (the Mg matrix) was reduced. As a result, micro-galvanic corrosion between the dispersed secondary phase and Mg matrix became weaker [36,37]. On the other hand, it has been suggested that fine dispersion of secondary-phase particles accelerated the activity of microgalvanic cell and decreased the corrosion resistance of Mg alloy fabricated via powder metallurgy route [38,39].

The controversy over the effect of finely dispersed secondary-phase particles on the corrosion resistance of Mg alloy exists and needs to be clarified. Microstructural parameters corresponding to different Mg alloys in the present work and in the references [36,37,38,39] are summarized in Table 3. The following observations can be drawn from the table. (1) All of the structures of Mg matrix were refined in the alloys prepared by powder metallurgy route. However, the degree of grain refinement was different from one to another. Strong refinements of Mg were found in the SWAPed AMX602 and MgRE_1.5_-PM alloy; while weak refinements of Mg were observed in WE43-PM and WZ21-PM350 alloys. Both weak and strong grain refinement of Mg were observed in the alloys having enhanced and reduced corrosion resistance. Thus, the refinement of Mg was not the main factor controlling the corrosion behavior. (2) Particle sizes of secondary phases varied from 700 nm to 50 nm. Compared to the size of secondary phases in the as-cast or extruded alloys, secondary-phase was refined. However, no correlation of particle size of secondary phase on corrosion resistance was observed. (3) Volume fractions of the secondary phase in the alloys prepared by powder metallurgy route, including EP4-Mg_97_Zn_1_Y_2_, SWAPed AMX602 and WE43-PM were dramatically reduced. Theses alloys showed enhanced corrosion resistance than their as-cast or extruded counterparts. In contrast, volume fractions of the secondary phases in WZ21-PM350 and MgRE_1.5_-PM, which showed decreased corrosion resistance, were very close to those in the as-cast or extruded ones. It is concluded that the volume fraction of secondary phase is the dominated factor that controls the corrosion resistance rather than those reported in reference [36,37,38,39].

In the present study, the as-cast Mg_97_Zn_1_Y_2_ contained *α*-Mg matrix with a lager crystallite size of 59 nm and a LPSO-Mg_12_Zn_1_Y_1_ phase distributed discontinuously along the grain boundary. The EP4-Mg_97_Zn_1_Y_2_ had finer *α*-Mg with a finer crystallite size of 41 nm, a super saturated solid solution having a higher Y content of 4.3 at % and finely dispersed Mg_24_Y_5_ particles. The volume fraction of Mg_12_Zn_1_Y_1_ phase in the as-cast sample was 17%, while the fractions of the Mg_24_Y_5_ in the EP4 sample was 4.7 wt %. The decrease in the fraction of secondary phase and increase in Y amount in the *α*-Mg matrix contributed to the enhancement of corrosion resistance of EP4-Mg_97_Zn_1_Y_2_. Both of the Mg_12_Zn_1_Y_1_ and Mg_24_Y_5_ were nobler than Mg matrix and they acted as cathodes in the microgalvanic cell. The fraction of secondary phase was smaller in the EP4 sample, which made microgalvanic corrosion weaker. It is well known that the increased presence of rare earth elements in magnesium matrix helped in the formation of more protective surface layers [40,41]. Y content was higher in the *α*-Mg matrix in the EP4 sample, which improved the passivity of surface film.

## 4. Conclusions

The bulk Mg_97_Zn_1_Y_2_ alloy was prepared by compacting mechanically-milled powders using equal channel angular pressing. Mechanical properties and corrosion behavior of the compacted alloy were investigated. The following conclusions can be made from the present research:

(1) The microstructure of the as-cast Mg_97_Zn_1_Y_2_ alloy consisted of *α*-Mg matrix and LPSO phase distributed discontinuously along the grain boundary. The ECAP-compacted bulk alloy contained *α*-Mg matrix and finely dispersed Mg_24_Y_5_ phase. From DSC analysis, it was possible that small amount of retained LPSO phase existed in the ECAPed alloy.

(2) The compacted alloy exhibited a hardness of 120 HV and a compressive yield strength of 308 MPa, which were higher than those of the as-cast Mg-Zn-Y alloy.

(3) The enhanced corrosion resistance observed in the ECAP-compacted alloy was attributed to the reduced volume fraction of secondary phase resulting in lower microgalvanic corrosion in the compacted alloy. The increase in Y content in the *α*-Mg matrix also contributed to the improvement of corrosion resistance.

## Figures and Tables

**Table 1:** Chemical composition of selected areas in the Mg_97_Zn_1_Y_2_ alloy under different processing states.

Processing State	Zone		Element	
		Mg (at %)	Zn (at %)	Y (at %)
As-cast	1	98.32 ± 0.26	0.58 ± 0.18	1.10 ± 0.22
	2	89.17 ± 0.75	4.62 ± 0.36	6.21 ± 0.45
5 h-milled	1	97.03 ± 0.63	1.18 ± 0.25	1.79 ± 0.49
	2	93.98 ± 1.79	1.15 ± 0.83	4.87 ± 1.13
	3	84.80 ± 1.50	1.15 ± 0.23	14.05 ± 1.54
4 passes of ECAP	1	96.29 ± 1.27	1.41 ± 0.17	2.30 ± 1.37
	2	94.45 ± 1.05	1.24 ± 0.14	4.31 ± 0.97
	3	84.42 ± 2.78	1.37 ± 0.49	14.21 ± 2.77

**Table 2:** XRD-calculated crystallite size of Mg and average particle size of Mg_97_Zn_1_Y_2_ alloy under different processing states.

Processing State	Time or Pass	Crystallite Size (nm)	Particle Size (μm)	Density (g/cm^3^)	Porosity (%)	Vickers Microhardness (HV)
As-cast	-	59 ± 9	-	1.87	-	80 ± 6
As-milled	5 h	24 ± 2	38	-	-	-
	10 h	23 ± 2	42	-	-	-
	15 h	24 ± 3	37	-	-	-
	20 h	22 ± 2	33	-	-	-
ECAP	1 pass	57 ± 3	-	1.76	4.6	123 ± 4
	2 pass	50 ± 2	-	1.77	4.0	122 ± 4
	4 pass	41 ± 1	-	1.80	2.4	121 ± 3

**Table 3:** Microstructural parameters corresponding to different Mg alloys in the present work and in the references [36,37,38,39].

Alloy	Processing Route	Secondary Phase	Grain Size of Mg (μm)	Particle Size of Secondary Phase (nm)	Volume Fraction of Secondary Phase (%)	Reference
As-cast Mg_97_Zn_1_Y_2_	Cast	LPSO	59 nm ^1^	-	17	Present work
EP4-Mg_97_Zn_1_Y_2_	Cast + MM + ECAP	Mg_24_Y_5_	41 nm ^1^	200 to 700	4.7	Present work
As-cast AMX602	Cast	Al_2_Ca	>50	-	5.4 ^2^	[36]
SWAPed AMX602	Cast + SWAP + Extrusion	Al_2_Ca	<1	<100	0.9 ^2^	[36]
WE43-IM	Cast + Extrusion	Mg_14_Nd_2_Y	0.5 to 4	-	19.7 ^2^	[37]
WE43-PM	Cast + Atomization + Extrusion	Mg_14_Nd_2_Y, Mg_24_Y_5_, Mg_45_Nd_5_	1 to 2	<50	10.7 ^2^	[37]
WZ21-IM350	Cast + Extrusion	LPSO	5	-	15.6 ^2^	[38]
WZ21-PM350	Cast + Atomization + Extrusion	Mg_24_Y_5_	2	<200	12.5 ^2^	[38]
MgRE_1.5_	Cast + Extrusion	LPSO, Mg_12_RE, Mg_24_Y_5_	5 to 7	-	24	[39]
MgRE_1.5_-PM	Cast + Atomization + Extrusion	Mg_24_Y_5_	0.6	<1 μm	26	[39]

^1^ Crystallite size was given for the alloys investigated in the present work. ^2^ Volume fraction of secondary phase reported in references [36,37,38] was calculated by image analysis software using the micrographs in the references.
